# The Effects of Ischemic Preconditioning Supplementation on Endothelial Function: A Systematic Review and Meta-Analysis

**DOI:** 10.1155/2021/6690691

**Published:** 2021-07-26

**Authors:** Xufang Gu, Zhichao Liu, Shengwei Gao, Li Ma, Jinhong Chen, Zhenxing Wang, Anmin Lu, Zhizhong Wang, Baohe Wang, Yuhong Li

**Affiliations:** ^1^The Second Affiliated Hospital of Tianjin University of Traditional Chinese Medicine, Tianjin, China; ^2^Tianjin University of Traditional Chinese Medicine, Tianjin, China

## Abstract

**Objective:**

Ischemic preconditioning (IPC) has gradually been promoted in clinical practice to lower the risk of cardiovascular surgery and postoperative complications. We investigated the role of IPC on vascular endothelial function and the relationship between IPC, flow-mediated dilation (FMD), and brachial artery diameter (BAD).

**Methods:**

Systematic searches were conducted in PubMed, Medline, Cochrane Library, Embase, and Scopus databases from their inception to March 20, 2020. This research included randomized controlled trials (RCTs) with adults, and the values of FMD and BAD were considered as the primary outcomes. Ten studies comprising 292 participants were included in the meta-analysis.

**Results:**

Regarding FMD, we observed beneficial effects of IPC on endothelial function (standardized mean difference (SMD): 1.82; 95% confidence interval (CI): 0.64, 3.01; *p* < 0.001; *I*^2^ = 89.9%). However, the available evidence did not indicate that IPC affected BAD (SMD: 0.08; 95% CI: −0.03, 0.18; *p* > 0.05; *I*^2^ = 76.5%).

**Conclusions:**

Our meta-analysis indicated a significant effect of IPC on the endothelial function of the blood vessels, affecting FMD but not BAD.

## 1. Introduction

Currently, approximately 120 million people in the US have different forms of cardiovascular disease (CVD), which is considered to be the leading cause of death, morbidity, and disability. In 2016, CVD accounted for about 840,000 deaths in the United States, and the number of CVD deaths increased from 2011 to 2017 by 9.7% [1]. The loss of function of the endothelium is considered to be an early pathogenic step in the development of atherosclerotic lesions and the subsequent onset of cardiovascular diseases [2]. Therefore, the endothelium has been identified as a tractable physiological target for therapeutic interventions to reduce the risk of CVDs such as coronary heart disease, stroke, or atherosclerosis [3]. Endothelium, which lines the inside of the blood vessels, regulates vascular integrity, reduces thrombosis, decreases vascular tone, improves vascular wall function, and promotes angiogenesis by releasing distinct signaling molecules [4, 5]. Moreover, the dysfunction of endothelium causes multiple diseases such as pathogenesis and progression of atherosclerosis, cerebrovascular disease, and inflammatory diseases [6]. Thus, the assessment of endothelial function could predict future cardiovascular events and provide an appropriate marker for blood vessels.

Nowadays, some noninvasive techniques have been developed to assess endothelial functions, such as FMD, which represents an endothelium-dependent, primarily nitric oxide- (NO-) mediated dilation of conduit arteries in response to an imposed increase in blood flow and shear stress. Moreover, impaired FMD has been associated with the predisposition to atherosclerosis and CVD and represents an early process in the development of target organ damage and clinical events [7]. In the 1990s, high-frequency ultrasonographic imaging of the brachial artery was developed to assess endothelium-dependent FMD. This technique stimulates the release of nitric oxide, resulting in vasodilation that can be quantitated as an index of vasomotor function [8].

IPC, originally proposed by Murry and his group in 1986, is associated with the ability of endogenous mechanisms to produce strong resistance to ischemic damage shortly after nonlethal mild ischemia or reperfusion treatment [9]. Animal studies [10, 11] have confirmed that IPC reduces ischemia-reperfusion injury in multiple organs. Much time and effort have been devoted to exploring the underlying molecular mechanisms of IPC. The most noteworthy result was that clinical research paid more attention to applying IPC for preventing distal organ damage. IPC was shown to limit the deleterious effects of prolonged ischemia or ischemia/reperfusion (IR), such as complex cardiac surgery, resection of abdominal aortic aneurysm, and kidney transplant operation, particularly in high-risk surgical patients. Many clinical studies have shown that IPC provided significant protection to the cardiac and vascular systems, improving the microcirculation state of blood vessels and maintaining endothelial function. Thus, IPC is expected to be an important therapeutic strategy to alleviate IR injury of vital organs in the future. However, FMD was calculated as a relative percentage change in the baseline BAD during reactive hyperemia; baseline BAD was also regarded as an important determinant of FMD [12].

Many clinical studies have analyzed the effects of IPC on endothelial function. Remote IPC before cardiac surgery increased myocardial salvage and protected endothelial function; additionally, it was safer and more economical than other alternatives [13]. Several studies have shown that IPC by transient limb ischemia reduces myocardial IPC injury in patients [13–17]. However, few studies presented protective effects of IPC against endothelial IR injury in patients who had suffered heart failure [18].

This study was conducted to systematically summarize the pieces of evidence for the effects of IPC on endothelial function and conduct a meta-analysis. In this study, two key indicators, FMD and BAD, were evaluated before and after ischemic treatment, and the effects of IPC on endothelial function were systematically examined for the first time in this study. We also evaluated FMD and BAD to further determine the interrelationships between the brachial artery variables and the cardiovascular risk events in a large well-characterized population.

## 2. Methods

Our systematic review was conducted according to the guidelines of the Preferred Reporting Items for Systematic Review and Meta-Analyses (Supplementary Table: PRISMA-P) [19], and the study protocol was registered with the identification code PROSPERO:CRD42020176093.

### 2.1. Data Sources and Search Strategy

Five databases (PubMed, Medline, Embase, Cochrane, and Scopus) were used to search for articles from inception until March 20, 2020. Additionally, a manual search of the list of references was performed for the relevant reviews and articles included in the systematic review. A search strategy was developed using the following MeSH and text keywords: intervention (“ischemic preconditioning” or “remote ischemic preconditioning” or “ischemic”) and outcomes (“FMD” or “BAD” or “resting diameter” or “brachial artery flow-mediated dilation” or “vasodilation” or “vascular reactivity” and “endothelial function”).

### 2.2. Literature Selection

Original studies were included if they met the following inclusion criteria: (1) relevant human intervention studies (subjects ≥18 years old), (2) performed IPC, (3) had a control group, and (4) measured endothelial function, including FMD and BAD.

Studies were excluded when (1) they had no information on the intervention or a control group, (2) duplicate publications or substudies of the RCTs were selected, (3) the studies were observational with cross-sectional, case-control, or cohort design, (4) the studies lacked sufficient BAD or FMD information, baseline, or follow-up, and (5) the studies were published in languages other than English.

### 2.3. Data Extraction

Two independent researchers screened the retrieved articles for eligibility. First, the title and abstract of all the studies were reviewed. Then, the full text of relevant studies was retrieved and assessed to ascertain the suitability of the study for inclusion in the meta-analysis. Any disagreement was discussed and resolved by the third researcher. After data extraction, the following information was recorded in a database: first author's name, publication year, sex, sample size, study design, intervention, duration of the study, and the mean and standard deviation for FMD and BAD in every intervention group and control group. Then, a random-effects meta-analysis was conducted, followed by meta-regression and subgroup analyses to determine whether the effects were modified by health status (i.e., healthy participants versus participants with other diseases), age, gender, ethnicity, and treatment duration.

### 2.4. Quality Assessment

The quality of the studies was assessed by two independent investigators using the Cochrane Collaboration risk of bias tool and met the following criteria: “random sequence generation, allocation concealment, blinding of participants and personnel, blinding of outcome assessment, incomplete outcome data, selective reporting, and other bias.” Based on the recommendations of the Cochrane Handbook, a decision of “yes” indicated a low risk of bias, while “no” indicated a high risk of bias. Labeling an item as “unclear” suggested an unclear or unknown risk of bias [19, 20]. Any disagreement was discussed and resolved by the third investigator.

### 2.5. Statistical Analysis

All statistical analyses for our meta-analysis were performed by the open-source statistical software Review Manager (RevMan, Version 5.3.5; The Nordic Cochrane Centre, The Cochrane Collaboration, Copenhagen, Denmark) and Stata version 15 (Stata Corp LLC, Texas, USA). Inverse variance weighting was used to pool the different studies [21]. Potential sources of heterogeneity were investigated by stratified meta-analyses according to various study characteristics defined a priori: year, characteristics, different interventions, and duration of intervention. Heterogeneity in the results was quantified by the *I*^2^ statistic [22]. Sensitivity analyses were performed to assess the robustness of the meta-analysis by removing one study at a time. Publication bias was assessed by visual inspection of the funnel plot [23] and Egger's regression test. The “trim and fill” method by Duval and Tweedie was used to adjust the analysis for the effects of publication bias [24].

## 3. Results

### 3.1. Study Characteristics

**Figure 1:**
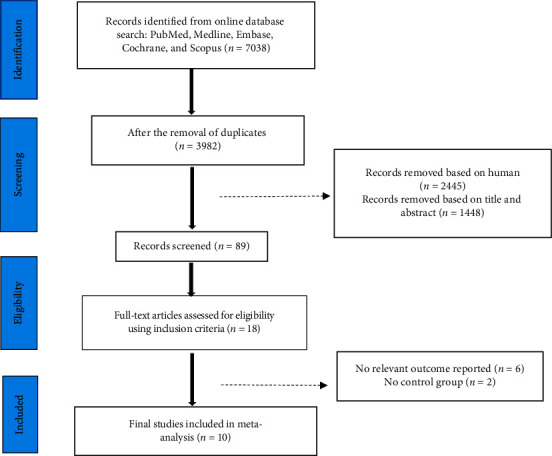
Flowchart showing the literature searched and reviewed for the selection of the studies.

The literature search identified 7,038 articles. Ultimately, ten studies with 12 trials were included in our research. A flow diagram of the process of selecting the studies is shown in Figure 1, and the details of the included studies are presented in Table 1.

In total, 292 participants were listed. The studies included were conducted in America, Europe, and Asia. The intervention period in these studies ranged between one day and eight weeks. The values of FMD and BAD were considered as outcomes.

### 3.2. Quality Assessment and Potential Bias

**Figure 2:**
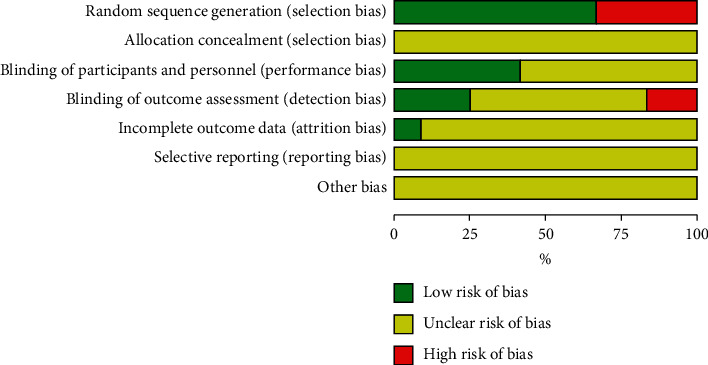
Summary of the risk of bias.

The quality score and risk of bias for each study are shown in Figure 2. The outcome of the quality assessment is provided in Table 1. All studies were randomized [18, 25–33], and four of the studies had additionally conducted before and after randomized trials [18, 33]. While it was hard to blind researchers and participants to the IPC protocol order, blinding the assessment of outcomes was performed in five studies [26–30]. Distribution concealment and reporting bias were not mentioned in any of the studies, which might have produced certain types of bias.

### 3.3. Effects of IPC on FMD

**Figure 3:**
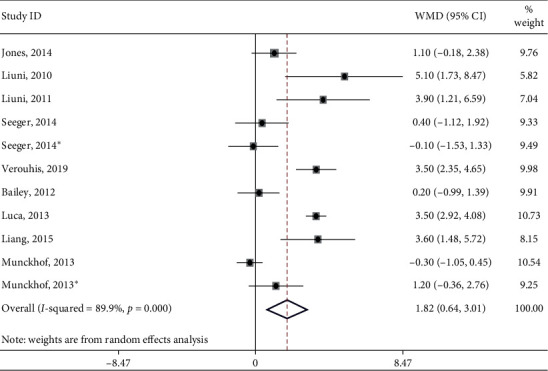
Forest plot showing the overall effect of IPC on flow-mediated dilation in adults (expressed as a percentage change). Data are shown as the percentage differences in means. Horizontal lines denote 95% CI. The size of the boxes is proportionally scaled to the effect size for each study.

Meta-analysis of the 12 sets of independent results showed that IPC improved endothelial function (SMD: 1.824; 95% CI: 0.64, 3.01; *p* < 0.05, shown in Figure 3). Heterogeneity between studies was significant (*Q* = 99.13; *I*^2^ = 89.9%; *p* < 0.05). The results indicated that IPC had a positive effect on the improvement of vascular function. The IPC group increased FMD by 1.82 compared to the FMD in the untreated group. Given that the methods for testing macrovascular and microvascular endothelium-independent reactivity remained largely unstandardized, meta-regression was only performed on the FMD data. Then, we performed subgroup analysis. The remaining heterogeneity after subgroup meta-analyses (Table 2) showed that age, gender, health status, ethnicity, and treatment duration might be responsible for the substantial amount of heterogeneity among the studies.

### 3.4. Effects of IPC on BAD

**Figure 4:**
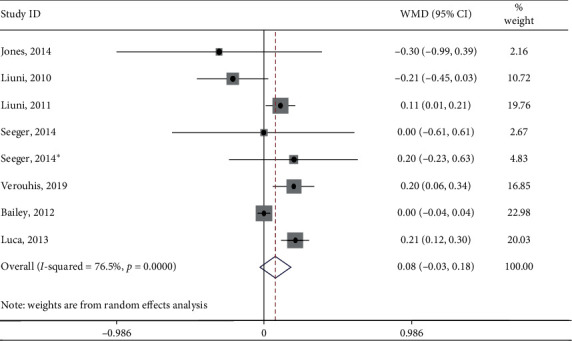
Forest plot showing the overall effect of IPC on BAD. Horizontal lines denote 95% CI. The size of the boxes is proportionally scaled to the effect size for each study.

Meta-analysis of the eight sets of independent results showed that IPC did not affect changes in BAD (SMD: 0.08; 95% CI: −0.03, 0.18; *p*=0.148, shown in Figure 4). Heterogeneity between studies was significant (*Q* = 29.73; *I*^2^ = 76.5%, *p* < 0.001), which was due to the variations in the characteristics of the populations (age, gender, or health status), different procedures and methods used (measurements of vascular function and intervention duration), and differences in the study design and quality of research. However, the sources of heterogeneity could not be fully determined since the number of included studies and the sample size of the majority of the studies were relatively small.

### 3.5. Sensitivity Analyses and Publication Bias

**Figure 5:**
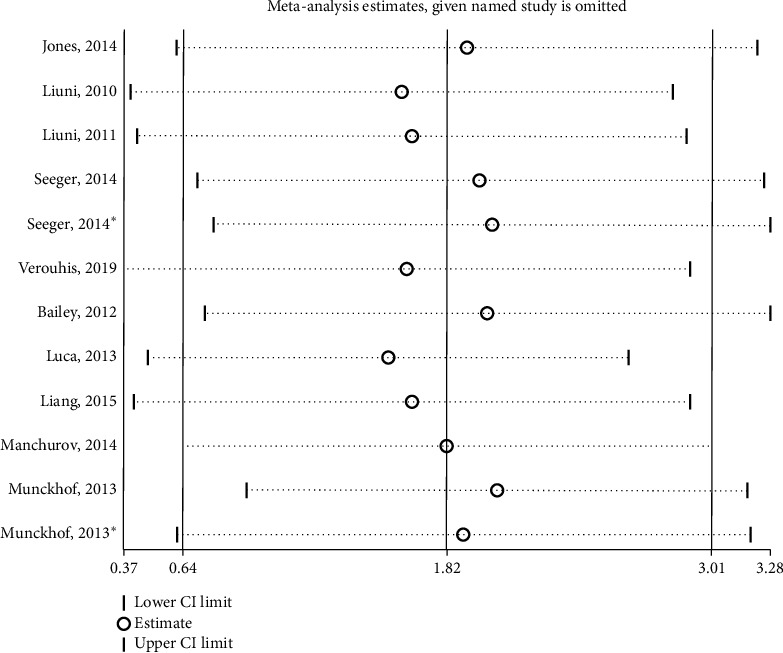
Sensitivity analyses of FMD.

**Figure 6:**
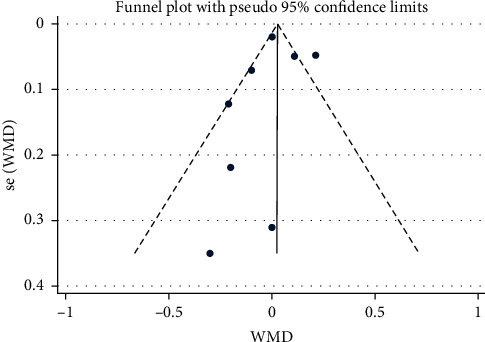
Funnel plot used to evaluate publication bias.

**Figure 7:**
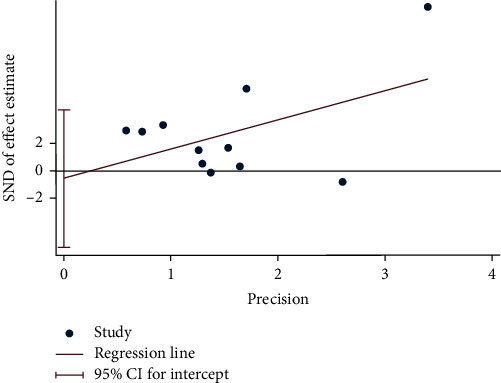
Egger's test used to evaluate publication bias.

The sensitivity analyses of FMD with the random-effects models are shown in Figure 5. Removing studies individually did not substantially modify the differences in the effect on FMD. Visual inspection of the funnel plot (Figure 6) suggested that, overall, there was no evidence of publication bias, which was also confirmed by Egger's Regression test (*p*=0.813; Figure 7) and Beeg's test (*p*=0.35).

## 4. Discussion

Overall, the results of the meta-analysis demonstrated that IPC protected endothelial function and improved FMD. However, it did not show any significant increase in BAD following the application of IPC.

Murry et al. first described the phenomenon of IPC [9]; several studies had demonstrated that IPC could reliably provide myocardial protection [34, 35]. The breakthrough in the clinical applicability of preconditioning protection came with the discovery by Kharbanda et al. that transient limb ischemia provided cardiovascular protection in humans and animals [36, 37]. Besides, IPC used as an adjunct to primary percutaneous coronary intervention in patients with ST-elevation myocardial infarction improved long-term clinical outcomes [17].

Endothelial dysfunction is involved in the development of atherosclerosis, which precedes asymptomatic structural vascular alterations, as well as clinical manifestations of CVD. Endothelial function can be assessed noninvasively using the FMD technique. Therefore, we can improve FMD through IPC and indirectly reduce the risk of related diseases. As an emerging detection indicator, FMD is closely related to the occurrence and development of many diseases. Moreover, brachial artery FMD has been used independently to predict long-term adverse CV events in healthy subjects with no apparent heart disease in addition to being used for assessing some traditional risk factors [38]. Besides, Perri et al. [39] found that AF patients with low FMD were associated with an increased risk of CVE (cardiovascular events), suggesting that impaired artery dilation predisposes to atherosclerotic complications. Additionally, some researchers suggested that the combination of FMD and nitroglycerine-induced vasodilation measurements could more accurately predict cardiovascular events than by measuring vasodilation with nitroglycerine only [40]. One study on systemic lupus erythematosus showed that the accumulation of damage in patients was associated with a progressive loss of FMD, with preserved endothelium-independent vasodilation [41]. Han et al. [42] found that low baseline FMD in hyperuricemia patients was associated with a significantly increased risk of incident hypertension and that FMD could be used as one of the predictive factors of the risk of diseases.

There are several mechanisms through which FMD could improve endothelial function and might account for the beneficial effects observed in this study. First, cardiovascular protection provided by the early phase of IPC is mediated by the stimulation of receptors linked to protein kinase C (PKC) activation by adenosine, bradykinin, NO, and free radicals [43–45]. Recently, Kharbanda et al. [36] reported that IPC might help to reduce endothelial injury during ischemic reperfusion in humans. Subsequent studies in humans have confirmed that IPC decreased inflammatory reaction and improved endothelial function by these humoral mediators [46–48]. One possible mechanism by which repetition of IPC augments endothelial function is by stepping up the vascular shear stress resulting from increased blood flow. Acute or chronic increase in the shear stress stimulates the release of NO in the blood vessels [49]. Additionally, a steady increase in shear stress has been shown to cause functional and histological alterations of the vascular endothelium, resulting in enhanced vascular structure and function [50]. This beneficial change in the endothelium after the repetition of IPC also might contribute to the augmented forearm vascular response to ACh (acetylcholine) and the ACh-stimulated NO release. On the other hand, preconditioning stimulus did not directly alter the endothelial function but avoided endothelial dysfunction in both the conduit and resistance vessels in response to IR.

In conclusion, our findings indicated that IPC augmented the endothelial function through an increase in FMD. It is important to select an appropriate intervention that is effective in improving or augmenting endothelial function. IPC has the potential for improving endothelial function as a novel method for predicting and preventing cardiovascular diseases associated with endothelial dysfunction. Besides, the idea of providing significant myocardial protection with transient limb ischemia is highly attractive to clinicians because it only requires a blood pressure cuff [16]. As a simple, safe, and feasible therapeutic technique, which can easily be applied in the preoperative setting to patients with acute cardiac events, IPC may have the potential to reduce mortality. Although IPC is a part of the most powerful and reproducible phenomenon in cardioprotection, it has not been readily translated into routine clinical use because of methodological hurdles and limitations. Overall, our findings need to be demonstrated in a larger multicenter trial before IPC can be implemented extensively as adjunctive therapy in clinical settings [17].

## 5. Limitations

The overall quality of the studies included was the modest. The majority of the investigations did not allow blind participants to the intervention arm, and no study reported methods of allocation concealment. Additionally, given the small number of studies included in this review, our analysis might have been underpowered to detect differences in the effectiveness of interventions based on health status, type of measurement, and study design. Differences in the effect size among the included studies could have been affected by the coexistence of traditional CVD risk factors (e.g., hypertension, smoking habit, diabetes mellitus, obesity, and hyperlipidemia) and the concomitant treatments. Further investigation is needed to establish the applicability and safety of IPC in clinical populations. The consideration of the other factors related to changes in cardiovascular risk is also warranted.

## Figures and Tables

**Table 1:** Details of the included studies.

Author	Publication year	Male	Country	Sample size (control/intervention)	Age	Intervention	Duration	Presented data
Jones et al. [25]	2014	16	England	8/8	24	IPC or not	8 weeks	FMD, BAD
Liuni et al. [26]	2010	20	America	10/10	18–33	IPC or not	5 days	FMD, BAD
Liuni et al. [27]	2011	18	America	9/9	18–29	IPC or not	20 days	FMD, BAD
Seeger et al. [18]	2014	15	The Netherlands	15/15	67	IPC or not	7 days	FMD, BAD
Seeger et al.^*∗*^ [18]	2014	15	The Netherlands	15/15	65	IPC or not	7 days	FMD, BAD
Verouhis et al. [28]	2019	4	The Swedish	4/4	30.5	IPC or not	6 days	FMD, BAD
Bailey et al. [29]	2012	11	England	11/11	25	IPC or not	Immediate	FMD, BAD
Luca et al. [30]	2013	15	Canada	15/15	20–31	IPC or not	1 day	FMD, BAD
Liang et al. [31]	2015	20	China	20/20	64	IPC or not	20 days	FMD
Manchurov et al. [32]	2014	26	Russia	25/23	62	IPC or not	7 days	FMD
Munckhof et al. [33]	2013	15	The Netherlands	15/15	72	IPC or not	7 days	FMD
Munckhof et al.^*∗*^ [33]	2013	15	The Netherlands	15/15	22	IPC or not	7 days	FMD

^*∗*^From the same article.

**Table 2:** Subgroup analyses for the effects of FMD on the markers of endothelial function.

Trial characteristic	Meta-regression analysis		Subgroup analysis					
	*P* value	95% CI	Stratification variable	Number of effect sizes	Pooled WMD	95% CI	*P* value within subgroups	*I* ^2^ (%)
Age	0.16	(−5.29, 1.14)	≤35 y	7	2.41	(1.15, 3.68)	<0.001	84.4
			≥60 y	5	0.66	(−0.70, 2.01)	0.008	74.5

Gender	0.42	(−3.70, 1.82)	>50% male	10	2.20	(0.83, 3.56)	<0.001	90.8
			≤50% male	2	0.28	(−0.66, 1.21)	0.839	0

Health status	0.86	(−4.25, 4.90)	Asymptomatic	9	1.82	(0.47, 3.16)	<0.001	91.4
			Diseased	3	1.91	(−1.22, 5.04)	0.016	82.7

Ethnicity	0.05	(−0.45, 3.62)	American	3	3.56	(3.00, 4.12)	0.64	0
			European	8	0.85	(−0.21, 1.92)	<0.001	81.5
			Asian	1	3.60	(1.48, 5.72)		

Treatment duration	0.68	(−1.99, 1.40)	5 min	2	0.14	(−0.91, 1.18)	0.64	0
			15 min	3	3.56	(3.00, 4.12)	0.64	0
			20 min	7	1.45	(0.08, 2.83)	<0.001	86.6

## Supplementary Materials

Supplementary Materials Supplemental Table: PRISMA-P.
