# Supplementary material for: The Effects of Ischemic Preconditioning Supplementation on Endothelial Function: A Systematic Review and Meta-Analysis
Source: Evid Based Complement Alternat Med. 2021 Jul 26;2021:6690691. doi: 10.1155/2021/6690691 (PMC8328691; doi:10.1155/2021/6690691)
Supplement: Supplementary Materials — Supplemental Table: PRISMA-P. [file 6690691.f1.docx]

**Supplementary material**

**PRISMA-P**

| **Section and topic** | **Item No.** | **Checklist item** | **Reported on page # / Yes or No** |
| --- | --- | --- | --- |
| **Administrative information** | | |  |
| Title | The effects of ischemic preconditioning supplementation on endothelial function: a systematic review and meta‐analysis | | See line 1, Page 1 |
| Identification | 1a | Identify the report as a protocol of a systematic review | See line 18, Page 2 |
| Update | 1b | If the protocol is for an update of a previous systematic review, identify as such | NO |
| Registration | 2 | If registered, provide the name of the registry (such as PROSPERO) and registration number | See line 97, Page 5 |
| **Authors** |  |  |  |
| Contact | 3a | Provide name, institutional affiliation, and e-mail address of all protocol authors; provide the physical mailing address of the corresponding author | See line 3, Page 1 |
| Contributions | 3b | Describe the contributions of the protocol authors and identify the guarantor of the review | See line 297, Page 16 |
| Amendments | 4 | If the protocol represents an amendment of a previously completed or published protocol, identify as such and list the changes; otherwise, state the plan for documenting important protocol amendments | See line 305, Page 16 |
| **Support** |  |  |  |
| Sources | 5a | Indicate the sources of financial or other support for the review | See line 294-296, Page 16 |
| Sponsor | 5b | Provide the name of the review funder and/or sponsor | See line 294-296, Page 16 |
| Role of the sponsor or funder | 5c | Describe the roles of funder(s), sponsor(s), and/or institution(s), if any, in developing the protocol | See line 294-296, Page 16 |
| **Introduction** | | |  |
| Rationale | 6 | Describe the rationale for the review in the context of what is already known | See line 35, Page 3 |
| Objectives | 7 | Provide an explicit statement of the question(s) the review will address regarding the participants, interventions, comparators, and outcomes (PICO) | See line 12-29, Page 2 |
| **Methods** | | |  |
| Eligibility criteria | 8 | Specify the study characteristics (such as PICO, study design, setting, time frame) and report the characteristics (such as years considered, language, publication status) to be used as eligibility criteria for the review | See line 98-102, Page 3 |
| Information sources | 9 | Describe all the intended information sources (such as electronic databases, contact with study authors, trial registers, or other grey literature sources) with planned dates of coverage | See line 108-110, Page 2 |
| Search strategy | 10 | Present a draft of the search strategy to be used for at least one electronic database, including planned limits, such that it could be repeated | See line 102-106, Page 2 |
| **Study records** |  |  |  |
| Data management | 11a | Describe the mechanism(s) that will be used to manage records and data throughout the review | See line 119-120, Page 5 |
| Selection process | 11b | State the process that will be used for selecting studies (such as two independent reviewers) in each phase of the review (i.e., screening, eligibility, and inclusion in meta-analysis) | See line 117, Page 5 |
| Data collection process | 11c | Describe the planned method for extracting data from reports (such as piloting forms, done independently, in duplicate), and any processes used for obtaining and confirming data from investigators | See line 118-121, Page 5 |
| Data items | 12 | List and define all the variables for which data will be sought (such as PICO items, funding sources), any pre-planned data assumptions, and simplifications | NO |
| Outcomes and prioritization | 13 | List and define all the outcomes for which data will be sought, including prioritization of the main and additional outcomes, with rationale | See line 120-127, Page 6 |
| Risk of bias in individual studies | 14 | Describe the anticipated methods for assessing the risk of bias in individual studies, including whether this will be done at the outcome or study level, or both; state how this information will be used in data synthesis | See line 120-127, Page 6 |
| Data synthesis | 15a | Describe the criteria by which the study data will be quantitatively synthesized | See line 130-136, Page 6 |
|  | 15b | If the data are appropriate for quantitative synthesis, describe the planned summary measures, the methods of handling the data, and the methods of combining data from studies, including any planned exploration of consistency (such as I2, Kendall’s τ) | See line 130-136, Page 6 |
|  | 15c | Describe any additional analyses proposed (such as sensitivity or subgroup analyses, meta-regression) | See line 138-149, Page 6 |
|  | 15d | If quantitative synthesis is not appropriate, describe the type of summary planned | See line 138-149, Page 6 |
| Meta-bias(es) | 16 | Specify any planned assessment of meta-bias(es) (such as publication bias across studies, selective reporting within studies) | See line 213-215, Page 12-13 |
| Confidence in cumulative evidence | 17 | Describe how the strength of the body of evidence will be assessed (such as GRADE) | See line 172, Page 9 |
